# Supplementary material for: Phosphorylation tunes electrostatically driven protein-RNA interactions
Source: Nat Commun. 2026 Oct 1;17:11278. doi: 10.1038/s41467-026-77886-2 (PMC13720170; doi:10.1038/s41467-026-77886-2)
Supplement: Supplementary file 2 [file 41467_2026_77886_MOESM2_ESM.pdf]

nature portfolio

Corresponding author(s): Benjamin Schuler, Nina Hartrampf, Marie Synakewicz

Last updated by author(s): 2026-08-24

Reporting Summary

Nature Portfolio wishes to improve the reproducibility of the work that we publish. This form provides structure for consistency and transparency in reporting. For further information on Nature Portfolio policies, see our Editorial Policies and the Editorial Policy Checklist.

Statistics

For all statistical analyses, confirm that the following items are present in the figure legend, table legend, main text, or Methods section.

n/a	Confirmed	
☐	☒	The exact sample size (*n*) for each experimental group/condition, given as a discrete number and unit of measurement
☐	☒	A statement on whether measurements were taken from distinct samples or whether the same sample was measured repeatedly
☒	☐	The statistical test(s) used AND whether they are one- or two-sided<br>*Only common tests should be described solely by name; describe more complex techniques in the Methods section.*
☒	☐	A description of all covariates tested
☒	☐	A description of any assumptions or corrections, such as tests of normality and adjustment for multiple comparisons
☐	☒	A full description of the statistical parameters including central tendency (e.g. means) or other basic estimates (e.g. regression coefficient) AND variation (e.g. standard deviation) or associated estimates of uncertainty (e.g. confidence intervals)
☒	☐	For null hypothesis testing, the test statistic (e.g. *F*, *t*, *r*) with confidence intervals, effect sizes, degrees of freedom and *P* value noted<br>*Give P values as exact values whenever suitable.*
☒	☐	For Bayesian analysis, information on the choice of priors and Markov chain Monte Carlo settings
☒	☐	For hierarchical and complex designs, identification of the appropriate level for tests and full reporting of outcomes
☒	☐	Estimates of effect sizes (e.g. Cohen's *d*, Pearson's *r*), indicating how they were calculated

Our web collection on statistics for biologists contains articles on many of the points above.

Software and code

Policy information about availability of computer code

Data collection	SymPhoTime 64 (PicoQuant) was used for single-molecule data collection. Agilent OpenLab CDS v.2.5 was used for the collection of UHPLC data. MassLynx V4.2 SCN991 was used for the collection of LC-MS data.
Data analysis	Fretica, a custom add-on package for Mathematica v.14.3 (Wolfram Research) was used for the analysis of single-molecule fluorescence data and is available at https://github.com/SchulerLab. Agilent OpenLab CDS v.2.5 was used for the analysis of UHPLC traces. MestReNova v.14.1 Mnova MS Suite (Mestrelab Research S.L.) was used for the analysis of LC-MS data. APBS v.3.4.1 was used to calculate the electrostatic surface potential of SRSF1, and PyMOL Molecular Graphics System v.3.0.0 was used to display the structure.

For manuscripts utilizing custom algorithms or software that are central to the research but not yet described in published literature, software must be made available to editors and reviewers. We strongly encourage code deposition in a community repository (e.g. GitHub). See the Nature Portfolio guidelines for submitting code & software for further information.

Data

Policy information about availability of data

All manuscripts must include a data availability statement. This statement should provide the following information, where applicable:
- Accession codes, unique identifiers, or web links for publicly available datasets
- A description of any restrictions on data availability
- For clinical datasets or third party data, please ensure that the statement adheres to our policy

The data generated in this study are provided in the Source Data file.

Research involving human participants, their data, or biological material

Policy information about studies with human participants or human data. See also policy information about sex, gender (identity/presentation), and sexual orientation and race, ethnicity and racism.

Reporting on sex and gender	n/a
Reporting on race, ethnicity, or other socially relevant groupings	n/a
Population characteristics	n/a
Recruitment	n/a
Ethics oversight	n/a

Note that full information on the approval of the study protocol must also be provided in the manuscript.

Field-specific reporting

Please select the one below that is the best fit for your research. If you are not sure, read the appropriate sections before making your selection.

☒ Life sciences ☐ Behavioural & social sciences ☐ Ecological, evolutionary & environmental sciences

For a reference copy of the document with all sections, see nature.com/documents/nr-reporting-summary-flat.pdf

Life sciences study design

All studies must disclose on these points even when the disclosure is negative.

Sample size	Sample sizes were determined by the normal throughput of the instrumentation while ensuring that independent replicates were performed for statistical analysis. For single molecule FRET experiments, each measurement reflects usually more than 1,500 freely diffusing molecules involved. In cases, where measurements reflect less than 1,500 molecules this is explicitly discussed in the text. In all measurements, the sample size was found to satisfactorily describe the conformational distribution in the ensemble.
Data exclusions	Bursts arising from freely diffusing molecules with only a single fluorophore and with < 60 photons were excluded from analysis. For fitting of binding isotherms, data containing substantial contributions from aggregates were excluded. More details on Data exclusion can be found in the Methods section.
Replication	The overall reproducibility of the main experiments was confirmed by making multiple independent measurements, by using different samples/variants, and by measuring on different days. The number of experimental replicates are specified in the supplementary information.
Randomization	No randomization was used.
Blinding	Experiments were not blinded. No a priori knowledge could be assumed, thus no randomization was required.

Reporting for specific materials, systems and methods

We require information from authors about some types of materials, experimental systems and methods used in many studies. Here, indicate whether each material, system or method listed is relevant to your study. If you are not sure if a list item applies to your research, read the appropriate section before selecting a response.

Materials & experimental systems

n/a	Involved in the study	
☒	☐	Antibodies
☒	☐	Eukaryotic cell lines
☒	☐	Palaeontology and archaeology
☒	☐	Animals and other organisms
☒	☐	Clinical data
☒	☐	Dual use research of concern
☒	☐	Plants

Methods

n/a	Involved in the study	
☒	☐	ChIP-seq
☒	☐	Flow cytometry
☒	☐	MRI-based neuroimaging

Plants

Seed stocks	n/a
Novel plant genotypes	n/a
Authentication	n/a
