# Supplementary material for: Phosphorylation tunes electrostatically driven protein-RNA interactions
Source: Nat Commun. 2026 Oct 1;17:11278. doi: 10.1038/s41467-026-77886-2 (PMC13720170; doi:10.1038/s41467-026-77886-2)
Supplement: Supplementary file 3 [file 41467_2026_77886_MOESM3_ESM.pdf]

nature portfolio

Peer Review File

Phosphorylation tunes electrostatically driven protein-RNA interactions

Corresponding Author: Professor Benjamin Schuler

This file contains all reviewer reports in order by version, followed by all author rebuttals in order by version.

Version 0:

Reviewer comments:

Reviewer #1

(Remarks to the Author)
In the manuscript by Hartrampf and Schuler and colleagues, the authors use single molecule FRET to characterize and quantify interactions between an RNA binding protein, SRSF1, in both a domain-specific and phosphorylation-specific manner. SRSF1 interacts with RNA both through its two RNA binding domains, as well as through its low complexity, disordered tail; this tail is enriched in serines, which become sequentially phosphorylated, neutralizing the charge of the tail, to regulate SRSF1 function. They use various lengths and sequences of RNA (both containing the specific sequences recognized by the RNA binding domains) and different lengths of RNA. By labeling the RNA with donor and acceptor fluorophores, single molecule FRET monitors changes in the RNA conformation upon binding to full-length SRSF1, a fragment corresponding to the RNA binding domains, and a fragment corresponding to the disordered tail. While the RNA binding domains demonstrate the expected sequence selectivity, they found the tail does not show sequence specificity, but does bind more tightly to longer RNA sequences. As expected, enzymatic phosphorylation of the tail of full-length SRSF1 greatly reduces binding. The authors then investigated phosphorylation of the tail in isolation more thoroughly. Through the use of synthetic peptides with 1-4 phosphorylations at different sites and recombinant peptides with S-> E mutations to mimic the charge effects of phosphorylation, they found that the overall net change in charge of the tail was the most important factor in reducing binding, rather than the specific site of phosphorylation or mutation; moreover, two S-> E mutations, which recapitulate the charge effects of a single S phosphorylation, were equally effective as the actual phosphorylation.

The data in this paper is beautiful, the approach to decoupling signal from protein aggregates by FCS is clever, and the observation regarding the equivalence of 2 glutamic acids per phosphate is interesting. This equivalence may be limited to cases interactions driven almost purely by electrostatic interactions. The overall scope of the work is somewhat narrow and it seems more appropriate for a more specialized journal.

Reviewer #2

(Remarks to the Author)
In this manuscript, Synakewicz et al describe a biophysical study that provides an important quantitative framework for understanding how phosphorylation regulates electrostatically driven protein–RNA interactions in intrinsically disordered proteins. Using single-molecule FRET, the authors demonstrate that phosphorylation functions as an electrostatic rheostat that continuously tunes RNA affinity rather than simply acting as an on/off switch. The finding that binding free energy scales linearly with net charge provides a predictive physical principle with broad implications for RNA biology, phase separation, and signaling. I would like to recommend publication of this work in Nature Communications after revision in the following points.

1. According to the counterion condensation theory, the release of counterions upon formation of protein-nucleic acid complex can make a major contribution to the binding free energy, and the effect is mainly entropic (for example, see Privalov et al. [2010] Nucleic Acids Res 39, 2483; and Yu et al. [2020] Acc Chem Res 53, 1802). Eq. 1 does not account for the major entropic effects of counterions.

2. The assumption of pKa ~ 5.6 for phosphoserine may be crude for highly charged systems. Since the systems studied in this work are positively charged, it is likely that the pKa shifts toward a lower value. In this sense, the charge of -2 at pH 8 is

reasonable for phosphoserine in this system. But this effect of pKa shift should be discussed for clarification.

3. The linear dependence of deltaG on the domain net charge is interesting but may not be surprising, given what is known about DNA binding of poly-lysine peptides and other poly-cations (for example, a very old example described in Record et al. [1976] J Mol Biol 107, 145). The literature on similar studies should be discussed to clarify the uniqueness of the findings in the current work.

Reviewer #3

(Remarks to the Author)
In this manuscript, the authors employed a systematic approach to probe how phosphorylation electrostatically affects the strength of the RNA-SRSF1 interaction. Binding affinities for a large set of fluorescently labeled RNAs with the full-length SRSF1, RRM12, and RS variants were quantified via smFRET and modeling. Chemically synthesized and recombinantly expressed RS peptides were used to fine-tune net charges.

Scientifically, the linear dependency of binding affinity on the net charges of the RS domain, either from phosphorylated residues or phosphomimetics, is in line with several reports on varying IDPs binding to partners, including cited references 2,3 and 58. The large set of constructs in Figure 2c is impressive and provides solid support for the averaged change of -0.5 kbT per single charge for the SRSF1 system. In my opinion, this study would appeal to a wide range of researchers working on the biophysical characterization of IDPs and the role of electrostatics on biomolecular binding, as it would be interesting to probe these energetic changes per single charge, from either phosphorylated residues or phosphomimetics, for other IDP systems.

Technically, the use of subpopulation-FCS to distinguish monomeric and oligomeric species in determining binding isotherms is innovative and generalizable. The use of an automated-flow system for peptide synthesis is excellent.

My two main concerns are listed as follows.
1. The authors need to ensure the labeling of the dyes on RNA does not affect the binding. Measurements using independent methods, such as ITC or SPR, for one or two constructs that are not aggregation-prone at concentrations needed for these experiments, would be sufficient.
2. The authors used an adjustable parameter n in Eq. 2 to extract the bound fraction. How this parameter was determined for various constructs was not mentioned in the methods, and should be added for reproducibility. In addition, does this parameter vary in different constructs?

I also have the following minor points for the authors to polish this manuscript:
1. Labeling the number of molecules in Figure 1C.
2. The authors need to justify the preset uncertainty of 2 for N=1 measurements in Figures 1e and 1f. These uncertainties are much less than the standard deviation from N>1 measurements on the log axis, as well as in Table S2.
3. Would the 1s bin size used in separating aggregates in binning photos affect the single-molecule FRET efficiencies and related binding affinity?
4. Representative trajectories of immobilized molecules for 60-nt RNA with the RS domain should be added to the SI with the accompanying kinetic scheme used in fitting binding parameters.

Version 1:

Reviewer comments:

Reviewer #2

(Remarks to the Author)
In this revision, the authors have addressed the issues this reviewer pointed out for the initial manuscript. I recommend publication of this manuscript in Nature Communications as is.

Reviewer #3

(Remarks to the Author)
The authors addressed all my comments satisfactorily. The added methods for the new data were well-documented. I particularly appreciate the new titration experiment (Fig. S4) using the new dye pair Alexa Fluor 488 / Alexa Fluor 594, where the enhanced binding affinity can be reliably accounted for by the additional net charges from the dye pair.

Open Access This Peer Review File is licensed under a Creative Commons Attribution 4.0 International License, which permits use, sharing, adaptation, distribution and reproduction in any medium or format, as long as you give appropriate credit to the original author(s) and the source, provide a link to the Creative Commons license, and indicate if changes were made.
In cases where reviewers are anonymous, credit should be given to 'Anonymous Referee' and the source.
The images or other third party material in this Peer Review File are included in the article's Creative Commons license, unless indicated otherwise in a credit line to the material. If material is not included in the article's Creative Commons license and your intended use is not permitted by statutory regulation or exceeds the permitted use, you will need to obtain permission directly from the copyright holder.
To view a copy of this license, visit https://creativecommons.org/licenses/by/4.0/

RESPONSE TO REVIEWER COMMENTS

We thank the reviewers for the careful reading of our manuscript and the excellent and very helpful suggestions for further improvements of the paper. We address the comments point by point below (in blue) and have marked the corresponding changes in the manuscript in red.

Reviewer #1 (Remarks to the Author):

In the manuscript by Hartrampf and Schuler and colleagues, the authors use single molecule FRET to characterize and quantify interactions between an RNA binding protein, SRSF1, in both a domain-specific and phosphorylation-specific manner. SRSF1 interacts with RNA both through its two RNA binding domains, as well as through its low complexity, disordered tail; this tail is enriched in serines, which become sequentially phosphorylated, neutralizing the charge of the tail, to regulate SRSF1 function. They use various lengths and sequences of RNA (both containing the specific sequences recognized by the RNA binding domains) and different lengths of RNA. By labeling the RNA with donor and acceptor fluorophores, single molecule FRET monitors changes in the RNA conformation upon binding to full-length SRSF1, a fragment corresponding to the RNA binding domains, and a fragment corresponding to the disordered tail. While the RNA binding domains demonstrate the expected sequence selectivity, they found the tail does not show sequence specificity, but does bind more tightly to longer RNA sequences. As expected, enzymatic phosphorylation of the tail of full-length SRSF1 greatly reduces binding. The authors then investigated phosphorylation of the tail in isolation more thoroughly. Through the use of synthetic peptides with 1-4 phosphorylations at different sites and recombinant peptides with S-> E mutations to mimic the charge effects of phosphorylation, they found that the overall net change in charge of the tail was the most important factor in reducing binding, rather than the specific site of phosphorylation or mutation; moreover, two S-> E mutations, which recapitulate the charge effects of a single S phosphorylation, were equally effective as the actual phosphorylation.

The data in this paper is beautiful, the approach to decoupling signal from protein aggregates by FCS is clever, and the observation regarding the equivalence of 2 glutamic acids per phosphate is interesting. This equivalence may be limited to cases interactions driven almost purely by electrostatic interactions. The overall scope of the work is somewhat narrow and it seems more appropriate for a more specialized journal.

> We thank the reviewer for the positive feedback and for highlighting the data quality, the single-molecule analysis, and the implications of our results. We agree that the equivalence between one phosphoserine and two glutamates should not be generalized indiscriminately to all phosphorylation-dependent interactions. We have therefore revised the Discussion to state more explicitly that this rule of thumb may fail in cases when phosphorylation acts in the context of structured binding interfaces, well-defined binding pockets, or coupled folding and binding. However, our results provide an important physical principle that is expected to apply to many cases where charged disordered regions interact with nucleic acids without forming well-defined structure. Given the abundance of charged disordered regions in the cell, especially in nuclear proteins [1,2], the tuning of charge-driven interactions by the degree of phosphorylation is thus likely to be of broad relevance, as also emphasized by Reviewers 2 and 3. Delineating the quantitative role of phosphorylation in cellular regulation is thus an important research direction, and besides the conceptual insights that our work affords, the combination of advanced peptide synthesis for introducing multiple phosphoserine residues and the use of subpopulation-specific single-molecule spectroscopy constitute a methodological advance for enabling such studies even

on systems where complications with aggregation and phase separation have previously prevented quantitative investigation.

Reviewer #2 (Remarks to the Author):

In this manuscript, Synakewicz et al describe a biophysical study that provides an important quantitative framework for understanding how phosphorylation regulates electrostatically driven protein–RNA interactions in intrinsically disordered proteins. Using single-molecule FRET, the authors demonstrate that phosphorylation functions as an electrostatic rheostat that continuously tunes RNA affinity rather than simply acting as an on/off switch. The finding that binding free energy scales linearly with net charge provides a predictive physical principle with broad implications for RNA biology, phase separation, and signaling. I would like to recommend publication of this work in Nature Communications after revision in the following points.

We thank the reviewer for highlighting the importance of the physical principle we identified and its important implications for biomolecular systems.

1. According to the counterion condensation theory, the release of counterions upon formation of protein-nucleic acid complex can make a major contribution to the binding free energy, and the effect is mainly entropic (for example, see Privalov et al. [2010] Nucleic Acids Res 39, 2483; and Yu et al. [2020] Acc Chem Res 53, 1802). Eq. 1 does not account for the major entropic effects of counterions.

We agree with the reviewer that counterion release can be a major entropic contribution to the binding free energy, as shown in the pioneering work of Lohman & Record and many others (and including our own work for highly charged IDPs, e.g. [3-5]). However, classical Record/Lohman theory does not directly relate the binding free energy to the net charge of the ligand, which is what we focus on here; it primarily relates the binding free energy to the salt concentration via the number of counterions released. In a system like ours, the number of released ions is expected to be closely related to the number of ion pairs formed in the bound state, and our results suggest that the number of released counterions increases roughly linearly with net charge of the RS domain.

The key point of Eq. 1 is to provide a rationalization for the linear dependence of binding free energy on the net charge of the RS domain, even if some aspects of the model may be very simple. To avoid an overly simplistic representation in terms of a single average distance between the RNA and the RS domain charges treated effectively as two point charges, we have now modified the presentation of our quantitative description of the binding free energy as a function of RS domain charge (Eq. 1). The linear dependence we observe experimentally can also be interpreted in terms of an effective, conformationally averaged electrostatic potential of the RNA that acts on the RS domain. To clarify these points and mention the role of counterion release explicitly, we thus added the following statement (p. 8):

"An alternative view of electrostatically driven protein-nucleic acid association emphasizes counterion release upon binding [58]. Such effects are not treated explicitly in Equation (1), but they are included phenomenologically in the measured free energy. In a Record-Lohman-type linkage description, an approximately linear dependence on net charge at fixed salt concentration is expected if the apparent number of released ions varies approximately linearly with RS-domain net charge, as previously suggested based on the length dependence of polylysine peptides binding to DNA [58, 59]. Our results extend this picture to a biologically regulated fully disordered protein-RNA interaction where negative phosphoserine or glutamate residues are introduced

while the arginine residues remain present, and they provide a quantitative calibration of how phosphorylation and phosphomimetic substitutions tune affinity over several orders of magnitude."

2. The assumption of pKa ~ 5.6 for phosphoserine may be crude for highly charged systems. Since the systems studied in this work are positively charged, it is likely that the pKa shifts toward a lower value. In this sense, the charge of -2 at pH 8 is reasonable for phosphoserine in this system. But this effect of pKa shift should be discussed for clarification.

We thank the reviewer for pointing this out. Indeed, the proximity of multiple arginine residues would be expected to lower the pK_a of phosphoserine further, making a charge of -2 an even better approximation for the RS domain. To make this point explicit, we have modified our statement in the Results section accordingly:

"Based on a phosphoserine pK_a of ~ 5.6 [40], which is likely to be even lower given the proximity to positively charged arginine residues [41], each phosphorylation contributes a charge of -2 per phosphate to the net charge of the protein at pH 8. SRPK1 phosphorylation thus inverts the net charge of SRSF1 and the RS domain, resulting in values of -12 and -13 for pSRSF1 and RS-16xpS, respectively."

3. The linear dependence of deltaG on the domain net charge is interesting but may not be surprising, given what is known about DNA binding of poly-lysine peptides and other poly-cations (for example, a very old example described in Record et al. [1976] J Mol Biol 107, 145). The literature on similar studies should be discussed to clarify the uniqueness of the findings in the current work.

We thank the reviewer for this important point. We had included a reference to a related publication from Lohman and Record (Zhang et al. Biophys. J. 1999), but we now added Record et al 1976 and Latt and Sober 1967 to the relevant paragraph discussing this point. We agree that a near-linear dependence of binding free energy on the number of charged groups has a strong precedent in the classical literature on oligocation-nucleic acid interactions. We now clarify that our results are consistent with and extend this classical framework. The key distinction is that the earlier studies changed the number of positive charges in simple oligocationic ligands by varying their length, whereas our study changes the net charge of a naturally occurring, arginine-rich disordered protein domain by multisite phosphorylation or by phosphomimetic substitutions. In this case, the arginine residues that can contact the RNA remain present, while phosphorylation or glutamate substitutions introduce negative charges that can reduce RNA binding through direct RNA repulsion, intrachain charge compensation, altered conformational ensembles, and changes in counterion release. It was therefore not obvious a priori that the binding free energy would collapse onto a single linear relation with RS-domain net charge, nor that one phosphoserine would be quantitatively equivalent to two glutamates. We have modified the text accordingly and (as mentioned in the response to point 1 above) now state that the observed linearity is in line with classical oligolysine-nucleic-acid studies and Record-Lohman counterion-release theory, but that the novelty of our work lies in establishing this principle for a biologically regulated, fully disordered protein-RNA interaction and in providing a quantitative calibration of how phosphorylation and phosphomimetic substitutions tune affinity over several orders of magnitude.

Reviewer #3 (Remarks to the Author):

In this manuscript, the authors employed a systematic approach to probe how phosphorylation

electrostatically affects the strength of the RNA-SRSF1 interaction. Binding affinities for a large set of fluorescently labeled RNAs with the full-length SRSF1, RRM12, and RS variants were quantified via smFRET and modeling. Chemically synthesized and recombinantly expressed RS peptides were used to fine-tune net charges.

Scientifically, the linear dependency of binding affinity on the net charges of the RS domain, either from phosphorylated residues or phosphomimetics, is in line with several reports on varying IDPs binding to partners, including cited references 2,3 and 58. The large set of constructs in Figure 2c is impressive and provides solid support for the averaged change of -0.5 kbT per single charge for the SRSF1 system. In my opinion, this study would appeal to a wide range of researchers working on the biophysical characterization of IDPs and the role of electrostatics on biomolecular binding, as it would be interesting to probe these energetic changes per single charge, from either phosphorylated residues or phosphomimetics, for other IDP systems.

Technically, the use of subpopulation-FCS to distinguish monomeric and oligomeric species in determining binding isotherms is innovative and generalizable. The use of an automated-flow system for peptide synthesis is excellent.

> We thank the reviewer for recognizing the importance of the advances made in our work, including the conceptual conclusions regarding the role of charge interactions as well as the methodological aspects regarding subpopulation-specific analysis based on single-molecule spectroscopy and the use of advanced peptide synthesis.

My two main concerns are listed as follows.
1. The authors need to ensure the labeling of the dyes on RNA does not affect the binding. Measurements using independent methods, such as ITC or SPR, for one or two constructs that are not aggregation-prone at concentrations needed for these experiments, would be sufficient.

> The reviewer raises a very important point: the fluorophores may influence the observed affinities. Wherever possible, cross-validation with alternative methods is thus desirable, as we have done in the past for other highly charged IDPs (see e.g. Chowdhury et al. [4], Sottini et al. [6]). However, using alternative methods for measuring RNA affinities for SRSF1 or the RS domain is very challenging because of the aggregation/phase separation propensity of the system at the concentrations required for ensemble methods, issues that we manage to eliminate with our subpopulation-specific single-molecule approach (see Figs. S1 and S2). Hence, it was not feasible to perform ensemble experiments with unlabeled RNA at 200 mM NaCl; even the best-behaved variant, SUMO-RRM12, required 400 mM NaCl to be soluble at the protein and RNA concentrations required for ITC. SPR approaches are further complicated by the pronounced surface adhesion of Arg-rich IDPs. However, to assess the reviewer's concern regarding the effect of the fluorophores on binding affinity, we performed competition titrations of SUMO-RRM12 with unlabeled rU9-CA/GGA (Fig. S4a, shown below), which showed that the affinities of RRM12 for the labeled and unlabeled rU9-CA/GGA are within a factor of two, our generic estimate of uncertainty for quantifying K_D values (see below). These results indicate that at least the sequence-specific interactions of the RRMs are not affected by the fluorophores to an extent that would affect our conclusions.
>
> For full-length SRSF1 and the RS domain, even the competition approach was not feasible due to aggregation/microphase separation upon adding the required amounts of RNA. However, as an alternative way of assessing the influence of the dyes on binding, we can compare the effect of different dye pairs. For comparison with the rU19 RNA labeled with the FRET pair Cy3B/CF660R

(total net charge: -1) that we used throughout the study, we now additionally performed measurements using the AlexaFluors 488 & 594 as donor and acceptor (total net charge: -4). The RS domain binds the AlexaFluor-labeled RNA with a K_D of ~70 nM, a ~5.6-fold higher affinity than the Cy3B/CF660R-labeled RNA (Fig. S4b, shown below; Table S2). However, we need to take into account the difference in net charge of -3 between the two pairs of fluorophores. In fact, this increase in affinity for the Alexa dyes is remarkably close to the roughly 5-fold difference expected based on the change in binding free energy of 0.55 k_BT per charge ($e^{3 \times 0.55} \approx 5.2$). This result indicates that the dyes do influence the affinity, but the magnitude of the observed effect is expected solely based on their charge. (Note that we had taken the net charge of the dye pair also into account for our original analysis.) This observation highlights the dominant role of electrostatics for the interactions between the binding partners and the consistency of our affinity measurements. We have added the data for the competition titration and the dye comparison to the SI (Fig. S4, see below) and adapted the manuscript in the relevant sections.

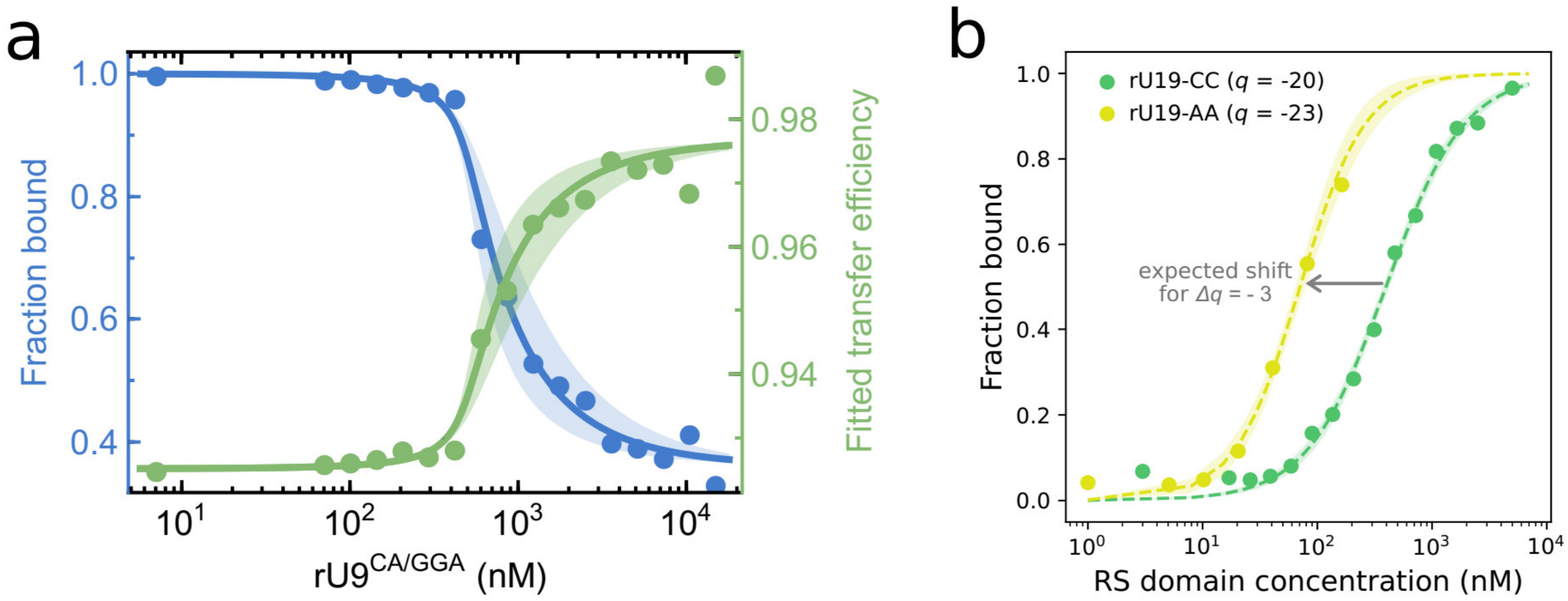

Fig. S 4. Quantifying the effect of fluorescent dyes on binding equilibria. (a) To assess the effect of fluorophores on RRM12-RNA interactions, labeled rU9-CA/GGA was out-competed with unlabeled rU9-CA/GGA. A global fit of the fraction bound and the average transfer efficiency to Equation (3) (for details see Methods), keeping the affinity of the labeled RNA fixed to ${}^{app}K_D^*$ = 20 nM, yields ${}^{app}K_D$ = 11 ± 2 nM for the unlabeled RNA (shaded bands indicate a variation in ${}^{app}K_D$ of a factor of 2). As this is within error of the direct titration, we conclude that the dyes have only minor effects on the sequence-specific interactions formed within the RRM12-RNA complex. (b) A competition with unlabeled RNA was not feasible in measurements of RS domain-RNA interactions due to aggregation/phase separation. Instead, we compared RS domain binding to rU19 RNA labeled with Cy3B/CF660R (CC, combined net charge added to the RNA: -1) and AlexaFluor488/AlexaFluor594 (AA, combined net charge added to the RNA: -4) to assess the effect of the fluorophores on affinity. The RS domain binds the AlexaFluor-labeled RNA with 5.6-fold higher affinity than the Cy3B/CF660R-labeled RNA. These results indicate that the dyes contribute to the binding interaction to the extent expected based on their net charge. Fit lines shown correspond to a fit to Equation (2) with 95% confidence intervals.

2. The authors used an adjustable parameter n in Eq. 2 to extract the bound fraction. How this parameter was determined for various constructs was not mentioned in the methods, and should be added for reproducibility. In addition, does this parameter vary in different constructs?

The adjustable parameter *n* was required in some cases to optimally describe the binding curves across the entire range of titrant concentrations, especially for SRSF1 and RS domain variants with the highest net charge, which are most prone to aggregation and (micro)phase separation. We thus included *n* as a free fit parameter for all binding curves. However, the midpoints of the binding isotherms, and thus the K_D values, are robust with respect to the choice of *n:* For all affinity measurements reported here, fits using Eqs. 2 or 4 with *n* set to 1 yielded K_D values within

a factor of 2 of the fits with n as a free parameter. We have now added this information in the Methods section (p. 13).

I also have the following minor points for the authors to polish this manuscript:

1. Labeling the number of molecules in Figure 1C.

Thank you for the suggestion — we have now included the numbers of observed molecules in the individual histogram panels.

2. The authors need to justify the preset uncertainty of 2 for N=1 measurements in Figures 1e and 1f. These uncertainties are much less than the standard deviation from N>1 measurements on the log axis, as well as in Table S2.

For most of our data, the factor of 2 as an uncertainty is more conservative than the standard deviations from repeated measurements. (We also note that the resulting error bars are asymmetric, whereas the estimates of the standard deviation from repeats are symmetric, probably contributing to the perceived lower uncertainty limit.) The uncertainty of about a factor of 2 (corresponding to a little less than $k_B T$ in free energy) captures the largest typical differences we observe between different protein and RNA preparations, between direct and competition titrations, and between different observables from the same experiment (e.g. binding curves obtained from transfer efficiency histograms, mean transfer efficiencies, diffusion times, or anisotropies). Hence, we quote this uncertainty for titrations for which we do not have duplicate measurements available. We have now improved the corresponding statement in the Methods section and also included a reference to Chowdhury et al. [4], where we previously used this estimate of the uncertainty in K_D.

3. Would the 1s bin size used in separating aggregates in binning photos affect the single-molecule FRET efficiencies and related binding affinity?

We thank the reviewer for making this point, which indicates a need for clarification. We only used the 1s binning in a first analysis step to identify and eliminate from the time traces fluorescence bursts caused by very large aggregates (i.e., species with much longer diffusion times than a single molecule). The resulting time traces were then used in a second step for the actual burst selection of single molecules, based on the interphoton time and a minimum threshold of the number of photons within each burst [7,8]. However, based on the FCS curves, we can still detect remaining, smaller oligomeric species or aggregates that cannot be removed from the trace by this two-step procedure. The corresponding data points were eliminated from the analysis as described in the workflow shown in Fig. S2. We have adapted the relevant Methods section (p. 12) to include this clarification.

4. Representative trajectories of immobilized molecules for 60-nt RNA with the RS domain should be added to the SI with the accompanying kinetic scheme used in fitting binding parameters.

As requested, we now provide a representative surface trajectory and the kinetic scheme used for analysis in Fig. S6 (shown below).

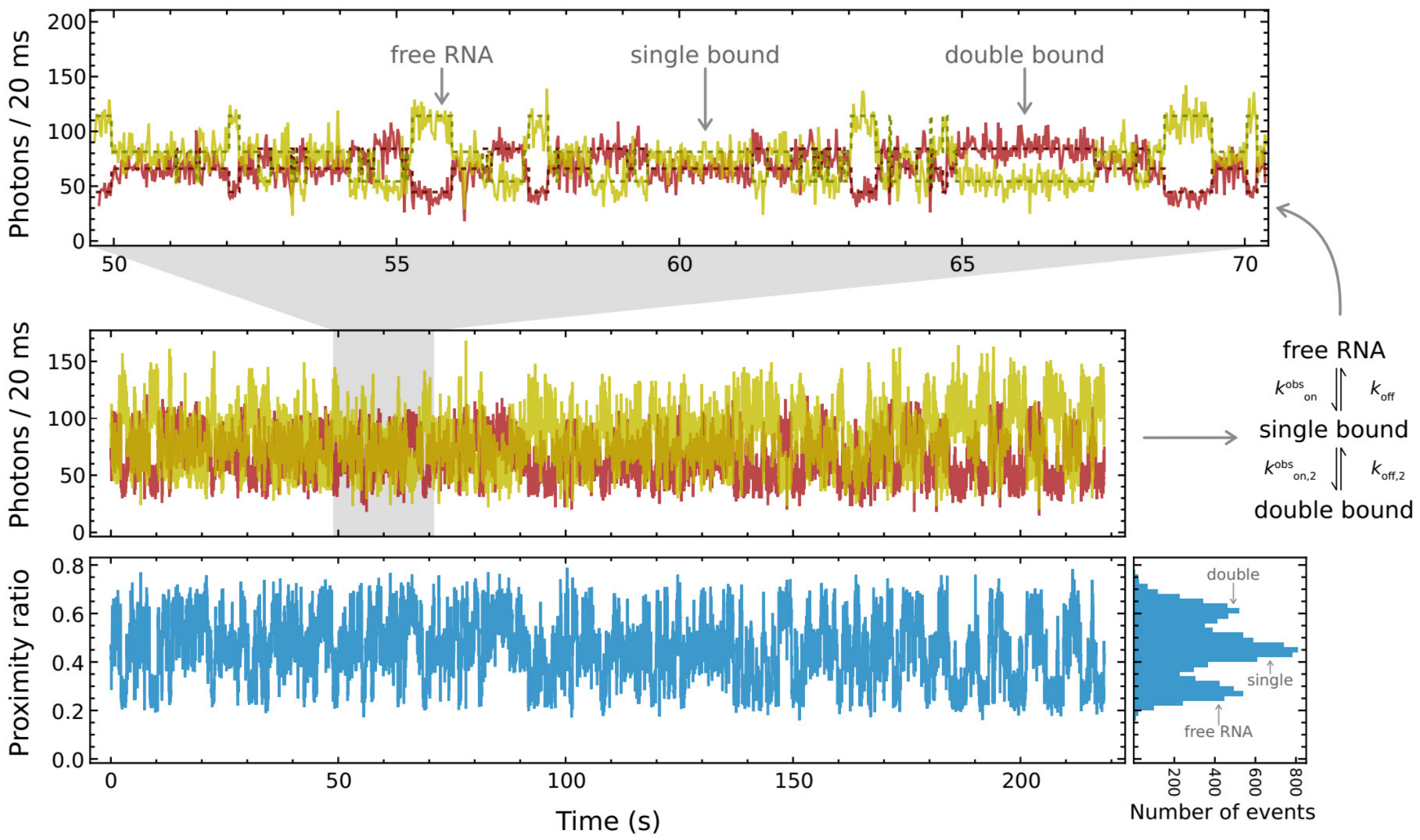

Fig. S 6 Representative binned photon count rate and proximity ratio time trace from experiments with surface-immobilized rU59-CA/GGA in the presence of 3.3 nM RS domain. Shown are the full trace with donor and acceptor signal colored in yellow-green and maroon, respectively, with a 20 s zoomed-in segment above, and the resulting proximity ratio (i.e. uncorrected transfer efficiency) below. For clarity, the most likely state trajectories (unbound/single bound/double bound) identified by the Viterbi algorithm are only shown in the zoomed picture. Rates were obtained from traces of 18 molecules with a combined total length of ~22 min. The maximum-likelihood analysis using Equation (8) yielded the following rates (uncertainties were estimated by bootstrapping): $k^{obs}_{on} = 0.8 \pm 0.2\ nM^{-1}s^{-1}$, $k_{off} = 1.3 \pm 0.2\ s^{-1}$, $k^{obs}_{on,2} = 0.6 \pm 0.1\ nM^{-1}s^{-1}$, $k_{off,2} = 4.4 \pm 0.5\ s^{-1}$.

References

1. Frege T, Uversky VN: **Intrinsically disordered proteins in the nucleus of human cells**. *Biochem. Biophys. Rep.* 2015, **1**:33-51.
2. Ruff KM, King MR, Ying AW, Liu V, Pant A, Lieberman WE, Shinn MK, Su X, Kadoch C, Pappu RV: **Molecular grammars of predicted intrinsically disordered regions that span the human proteome**. *Cell* 2026, **189**:323-342 e317.
3. Borgia A, Borgia MB, Bugge K, Kissling VM, Heidarsson PO, Fernandes CB, Sottini A, Soranno A, Buholzer KJ, Nettels D, et al.: **Extreme disorder in an ultrahigh-affinity protein complex**. *Nature* 2018, **555**:61-66.
4. Chowdhury A, Borgia A, Ghosh S, Sottini A, Mitra S, Eapen RS, Borgia MB, Yang T, Galvanetto N, Ivanovic MT, et al.: **Driving forces of the complex formation between highly charged disordered proteins**. *Proc. Natl. Acad. Sci. U.S.A.* 2023, **120**:e2304036120.
5. Holmstrom ED, Liu ZW, Nettels D, Best RB, Schuler B: **Disordered RNA chaperones can enhance nucleic acid folding via local charge screening**. *Nat. Commun.* 2019, **10**.
6. Sottini A, Borgia A, Borgia MB, Bugge K, Nettels D, Chowdhury A, Heidarsson PO, Zosel F, Best RB, Kragelund BB, et al.: **Polyelectrolyte interactions enable rapid association and dissociation in high-affinity disordered protein complexes**. *Nat. Commun.* 2020, **11**:5736.
7. Nir E, Michalet X, Hamadani KM, Laurence TA, Neuhauser D, Kovchegov Y, Weiss S: **Shot-noise limited single-molecule FRET histograms: Comparison between theory and experiments**. *J. Phys. Chem. B* 2006, **110**:22103-22124.
8. Eggeling C, Berger S, Brand L, Fries JR, Schaffer J, Volkmer A, Seidel CA: **Data registration and selective single-molecule analysis using multi- parameter fluorescence detection**. *J. Biotechnol.* 2001, **86**:163-180.
